# Maternal blood inflammatory marker levels increased in fetuses with ventriculomegaly

**DOI:** 10.3389/fnhum.2022.998206

**Published:** 2022-12-05

**Authors:** Qiang Li, Xin-Wei Ju, Jing Xu, Jiuhong Jiang, Chang Lu, Xing-Da Ju

**Affiliations:** ^1^School of Psychology, Northeast Normal University, Changchun, China; ^2^Department of Radiology, The First Hospital of Jilin University, Changchun, China; ^3^School of Life Sciences, Northeast Normal University, Changchun, China; ^4^School of Information Science and Technology, Northeast Normal University, Changchun, China; ^5^Jilin Provincial Key Laboratory of Cognitive Neuroscience and Brain Development, Changchun, China; ^6^Autism Centre of Excellence, Northeast Normal University, Changchun, China

**Keywords:** fetal ventriculomegaly, prenatal infection, inflammatory markers, neurodevelopment, biomarkers brain alterations

## Abstract

**Background:**

Fetal ventriculomegaly (VM) is one of the most common abnormalities of the central nervous system (CNS), which can be significantly identified by brain anomalies prenatally by magnetic resonance imaging (MRI). Aberrant white blood cells (WBCs) levels indicate that the maternal is suffering from the infection. Previous studies have confirmed that prenatal infection can affect fetal brain structure, but there is no research revealed the association between maternal blood parameters with fetal VM until now.

**Methods:**

We measured the width of the lateral ventricle of 142 fetuses, which were divided into the fetal VM group (*n* = 70) and the normal lateral ventricle group (*n* = 72). We compared maternal blood cell levels between the two groups and investigate potential biomarkers of fetal VM.

**Result:**

High levels of maternal WBC and neutrophil (NE#) levels were observed in fetuses with VM (*p* < 0.001), while lymphocyte percentage, monocytes (MO#), neutrophil/lymphocyte ratio (NLR), and platelet were also increased in the fetal VM group (*p* = 0.033, 0.027, 0.034, and 0.025, respectively). receiver–operator curve (ROC) analysis suggested that WBC and NE# counts might be useful to distinguish fetuses with enlarged lateral ventricles (AUC = 0.688, 0.678, respectively).

**Conclusion:**

The current study emphasizes the importance of maternal infection for fetal brain growth, which could provide important information for prenatal diagnosis of CNS anomalies. Future research needs longitudinal analysis and exploration of the influence of maternal blood inflammatory marker levels on fetal brain development.

## Introduction

Enlarged fetal lateral ventricle is one of the most common findings in fetal abnormal cerebral development which is linked to many potential central nervous system (CNS) abnormalities, with a reported incidence of approximately 1% (Fox et al., 2018). Fetal ventriculomegaly (VM) is the result of a defect in cerebrospinal fluid (CSF) circulation or resorption, and may also be caused by chromosomal abnormalities and intrauterine infections (Pisapia et al., 2017). Fetal VM is typically classified according to the degree of ventricular dilatation between 15 and 40 weeks of gestation, as mild (10–12 mm), moderate (12–15 mm), or severe (≥15 mm) (Fox et al., 2018). An increased risk of neurodevelopmental delay is positively associated with a higher degree of dilatation (Weichert et al., 2010). A prospective cohort study assessed the neurodevelopmental outcomes of 94 fetuses with VM, the rate of neurodevelopmental impairment of fetuses with mild and moderate VM increased from 8.3 to 17.6% (Sun et al., 2021).

Increased volume of ventricles are considered to represent the neurobiological basis of neurodevelopmental and psychiatric disorders, including autism spectrum disorder (ASD) and schizophrenia (Turner et al., 2016; Kuo and Pogue-Geile, 2019). Research has indicated abnormal brain structures are associated with the pathogenesis of ASD, and the most common abnormal brain structure types in autistic children were lateral VM (Zhang et al., 2019). More importantly, research has implicated that the abnormal enlargement of lateral ventricles could have occurred during prenatal brain development. A recent cohort study shows that fetuses diagnosed with VM were associated with an increased risk of neurodevelopmental disorders such as ASD, epilepsy, and impaired psychomotor development (Thorup et al., 2019). Notably, prenatal diagnosis may reduce the risk of moderate or severe impairments to 7% (Laskin et al., 2005). Therefore, it is necessary to discern abnormal lateral ventricular growth through meticulous examination during the prenatal stage, which may offer a means to identify fetuses with a high risk of neurodevelopmental disorders.

Magnetic resonance imaging (MRI) is the imaging technique of choice for identifying significant brain structural anomalies prenatally. Because of its good regional resolution, several studies have used MRI to measure brain growth and investigate fetal brain abnormalities, including VM, agenesis of the corpus callosum, posterior fossa anomalies, and malformations of cortical development (Masselli et al., 2020). In addition, MRI can observe subtle intracranial findings like cortical migration anomalies, intracranial hemorrhage, and neuronal migration, which is difficult to be detected by ultrasound (Di Mascio et al., 2020; Mirsky et al., 2020). Based on these findings, current practice suggests a prenatal MRI examination in the second or third trimester of pregnancy to help physicians accurately identify brain anomalies in a primary stage of development, and inform appropriate clinical treatment plans and parental counseling (Pisapia et al., 2017).

While fetal MRI is feasible, these scans are costly, and there are concerns about the potential adverse effects of the strong magnetic field on the developing brain (Tocchio et al., 2015). High static field exposure and high acoustic noise levels may have an underlying impact on the developing fetus (Mervak et al., 2019; Nagaraj et al., 2021). Notably, blood tests have many advantages as a necessary check for prenatal examination. First of all, unlike MRI, routine maternal blood tests can be accessed easily during the whole pregnancy process in clinical examination. In addition, maternal blood provides valuable information for the physiological status of patients or pregnant women, physiologic changes will directly affect hematologic parameters. Maternal complete blood counts (CBC) objectively measured the level of white blood cells (WBCs), red blood cells (RBCs), and platelet count (PLT). The variety of WBCs serve to protect the host from harm, they are also involved in disease pathogenesis and complications (Jackson and Miller, 2020). Thus, peripheral biomarkers of inflammation derived from CBC have been investigated in numerous studies and suggested to reflect the pathogenesis of diseases.

Establishing the relationship between hematological parameters and neurodevelopmental disorders has become an increasingly popular area of research. Several lines of evidence demonstrated elevated levels of WBCs in both schizophrenia and bipolar disorder (Liemburg et al., 2018; Kirlioglu et al., 2019). Maternal CBC may better understand the intrauterine environment and become a new biomarker of neurodevelopmental disorders. Early-life environmental insults such as infections change fetal brain structure and become a risk factor for neurodevelopment disorders (Estes and McAllister, 2016; Jones and Van de Water, 2019; Kreitz et al., 2020). Notably, the latest article demonstrated immune dysregulation in mothers with autistic offspring at the gestation period, and the result shows that the levels of plasma immunological markers in maternal gestational and child cord blood from autistic children change significantly (Che et al., 2022). The results of this study not only reveal the possibility of predicting autism in the embryonic stage but also provide robust evidence for the influence of prenatal infection on neurodevelopment. Furthermore, preliminary evidence noted strong associations between maternal raised levels of cytokines and neonatal brain abnormal structure. Higher maternal Interleukin-6 (IL-6) levels during pregnancy correlated with changes in brain structure and function, altered frontolimbic white matter, and cognitive development in early life (Rasmussen et al., 2019; Nazzari and Frigerio, 2020). Interestingly, infections lead to ventricular obstruction and maldevelopment, which are one of the etiological factors of fetal VM (Mirsky et al., 2020). However, there is still a lack of consensus about the association between maternal WBCs and fetal VM.

Most of the existing studies only use neuroimaging to understand the abnormal development of the fetal brain, but if we can combine the maternal WBCs and MRI as two effective indicators to evaluate fetal brain development, we can reduce the potential adverse effects of fetal MRI. To investigate potential associations between an altered immune environment and abnormal fetal brain structures, we examined maternal WBCs and other blood index levels in 70 fetuses with VM and 72 fetuses with normal ventricular. We try to provide novel evidence for the potential relationship between maternal WBCs and fetal VM. In the era of rapidly developing technologies, we believe that the results of this study will assist in evaluating the abnormal brain development in embryonic and inform current clinical management as well as parental counseling, particularly having important implications for the effective targeted intervention.

## Materials and methods

### Subjects

This retrospective study enrolled pregnant women who visited the First Bethune Hospital of Jilin University from January 2019 to May 2021. The exclusion criteria were as follows: (1) family history of neurological diseases; (2) fetus with abnormal chromosome; (3) pregnant women’s blood collection missed. During this period, 142 singleton fetuses met the inclusion/exclusion criteria. Among them, 70 fetuses with VM (an atrial width greater than 10 mm), and the remaining 72 cases have normal lateral ventricle width. Our study was approved by the First Bethune Hospital of Jilin University Research Ethics Committee (ethics no. 2021-704). The strengthening the Reporting of Observational Studies in Epidemiology (STROBE) statement was used as a reporting guideline for this study. The requirement of informed consent was waived due to the retrospective nature of the study.

### Fetal magnetic resonance acquisition

**FIGURE 1:**
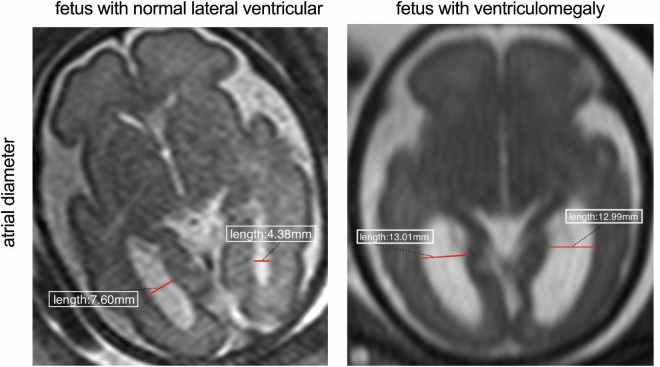
Comparison between fetus with normal lateral ventricular and wider ventricle. The red line indicates the width of the atrial diameter.

All participants in this study underwent fetal MRI. MRI scanning was performed using a 1.5T scanner (Avanto Siemens Medical Systems, Erlangen, Germany) using a 5-channel body phased array cardiac coil. The mother was positioned feet-first into the scanner without intravenous contrast or sedative premedication, and the total duration of the MR examination did not exceed 60 min. A complete fetal brain clinical examination was performed in transverse, sagittal, and coronal planes. T2-weighted half-Fourier acquired single-shot turbo-spin echo (HASTE) was acquired using the following scanning parameters: TR = 1350 ms, TE = 94 ms, slice thickness/gap were 4.80 mm/4 mm; field of view, 400 mm; matrix, 256× 256; flip angle = 90°, and acquisition time is 24 s and for true fast-imaging with steady precession (True-FISP) sequences were: TR = 337.25 ms, TE = 1.19 ms, section thickness/gap, 4 mm/0.4; field of view, 380; matrix, 320 × 255 flip angle, 90°; and sequence acquisition time of 29–45 s. The total scan time for the mother was 3–4 min. Acquired MRI images were input and obtained into the picture archiving and communication system (PACS) and measured the right and left atrial diameters were by experienced radiologists. The atrial diameter is measured on the transverse plane at the level of the atria and takes mid-height of the ventricle (with good visibility of the choroid plexuses) (Figure 1). According to previous studies reported, the present study used the same measurement method to measure the atrial diameter at the level of the atrium (Kyriakopoulou et al., 2017).

### Clinical variables

Demographic data (age and gestation age) were collected from the medical records available in the hospital. The fasting venous blood samples of pregnant women were collected in the morning after 8 h fasting. Sysmex XN-1000 automatic hematology analyzer (Sysmex, Kobe, Japan) was used to measure the complete blood count of pregnant women in the Bethune First Hospital of Jilin University. Treatment of blood samples within 30 min after blood collection. Monocyte-to-lymphocyte blood cell ratio (MLR), neutrophil-to-lymphocyte blood cell ratio (NLR), and platelet-to-lymphocyte blood cell ratio (PLR) of the participators was calculated by Microsoft Excel, version 16.

### Statistical analysis

Statistical analyses were carried out using SPSS 25.0 (SPSS Inc., Chicago, IL, USA) was used for analysis. The Shapiro–Wilk test was used to check normality in data distribution. Data are presented as mean (SD) for continuous variables. Medians [interquartile ranges (IQRs)] are used if the data are not a normal distribution. *T*-test was used for continuous variables with normal distribution. Comparisons between two groups of non-normally distributed independent variables were analyzed using the Mann–Whitney *U*-test. The correlations of fetal left and right laternal ventricle width and variables that present non-parametric distributions were calculated using Spearman’s Rank Correlation (presented as Spearman rho). The logical regression equation established the relationship between fetal VM and abnormal maternal blood cell levels. At first, we performed logistic regression separately for each analyte to predict fetal VM. Subsequently stepwise logistic regression model was used to explore optimal biomarkers. Only significant analytes from the analyses (*p* < 0.05) were made available for the selection of a candidate biomarker panel. The results are expressed as odds ratios (ORs) with the corresponding 95% confidence intervals (CIs). A receiver–operator curve (ROC) was used to assess the accuracy of maternal blood levels to diagnose fetal VM and the area under the curve (AUC) was used to calculate the measurements of the accuracy of the test. The optimal cut-off points of the maternal inflammatory markers were evaluated by the Youden index [maximum (sensitivity + specificity −1)]. Graphing was created using GraphPad Prism 9.0.

## Results

### Sample characteristics

**FIGURE 2:**
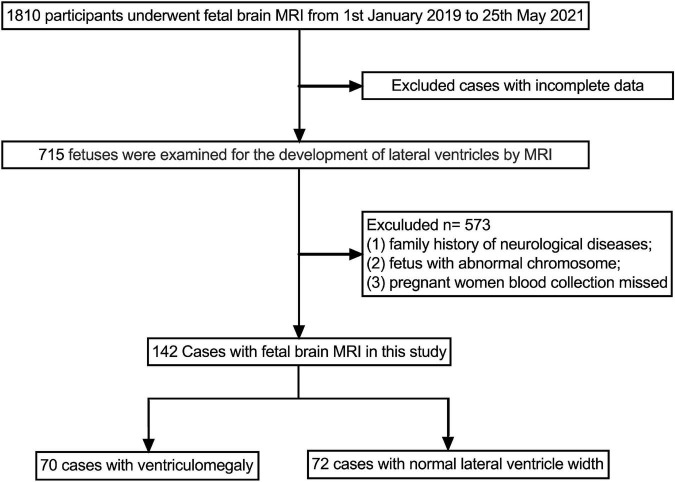
Flowchart study population.

A total of 142 participants were enrolled in this study, 70 cases were in fetal VM group, and 72 cases were in the normal ventricular group (Figure 2). The mean maternal age of the fetal VM group was 31 years (range = 21–39 years), and the gestational age was 23–37 weeks. The mean maternal age of fetuses with the normal lateral ventricular group was 30 years (range = 18–43 years), and the gestational age was 22–35 weeks. There was no significant difference between cases with or without VM in mean age (*P* = 0.576) or gestational age (*P* = 0.933). The Shapiro–Wilk test for normality suggested that the maternal ages, RBC, hemoglobin (HGB), hematocrit (HCT), and PLT were normally distributed (Table 1). Gestational ages (GA) and other maternal blood indicator analytes were non-normally distributed (Table 2). *T*-test also presented that there were no significant differences between fetal VM and fetuses with normal ventricle group in the number of the maternal RBC, HGB, or HCT (*P* > 0.05).

**TABLE 1:** Demographic features and maternal blood parameters in different groups.

Variables	Normal ventricular (*n* 72)	Ventriculomegaly (*n* 70)	*t*	*P*-value
MA (years)	30.580 ± 4.797	31.000 ± 4.014	–0.561	0.576
RBC (10^12^/L)	3.953 ± 0.405	3.940 ± 0.374	0.204	0.839
HGB (g/L)	122.600 ± 12.359	120.490 ± 11.418	1.057	0.292
HCT (%)	0.363 ± 0.035	0.360 ± 0.030	0.657	0.512
PLT (10^9^/L)	204.810 ± 55.070	227.13 ± 62.278	–2.264	0.025

Pregnancies ages and maternal peripheral blood levels in the normal ventricular group (*n* 72) and fetal ventriculomegaly (VM) group (*n* 70). ***P < 0.001, **P < 0.01, and *P < 0.05. *P*-values less than 0.05 were considered statistically significant. MA, maternal age; RBC, red blood cell; HGB, hemoglobin; HCT, hematocrit; PLT, platelet count.

**TABLE 2:** Comparison of maternal blood parameters in the normal ventricular group and fetal ventriculomegaly (VM) group.

Variables	Normal ventricular (*n* 72)	Ventriculomegaly (*n* 70)	*Z*	*P*-value
GA (weeks)	30.000 (27.000, 32.000)	30.000 (27.000, 32.000)	−0.084	0.933
WBC (10^9^/L)	9.230 (8.018, 10.050)	10.320 (9.248, 11.723)	−3.868	0.001
NE (%)	0.760 (0.730, 0.780)	0.770 (0.730, 0.800)	−1.820	0.069
LY (%)	0.170 (0.150, 0.198)	0.155 (0.130, 0.20)	−2.127	0.033
NE# (10^9^/L)	6.895 (5.640, 7.648)	7.870 (6.843, 9.260)	−3.664	0.001
LY# (10^9^/L)	1.525 (1.283, 1.863)	1.595 (1.380, 1.928)	−0.771	0.441
NLR	4.505 (3.705, 5.088)	4.965 (3.618, 6.215)	−2.124	0.034
MO# (10^9^/L)	0.540 (0.430, 0.628)	0.610 (0.488, 0.730)	−2.214	0.027
MLR	0.350 (0.260, 0.420)	0.360 (0.280, 0.480)	−1.186	0.236
PLR	131.220 (106.678, 159.518)	143.730 (105.188, 172.743)	−1.098	0.272
MPV (fL)	10.600 (10.125, 11.300)	10.500 (9.875, 11.125)	−1.140	0.254

Gestational ages (GA) and maternal peripheral blood levels in the normal ventricular group (*n* 72) and fetal ventriculomegaly (VM) group (*n* 70). ***P < 0.001, **P < 0.01, and *P < 0.05. *P*-values less than 0.05 were considered statistically significant. GA, gestational age; WBC, white blood cell; NE%, neutrophil percent; LY%, lymphocyte percent; NE#, neutrophil; LY#, lymphocyte; NLR, neutrophil/lymphocyte ratio; MO#, monocytes; MLR, monocyte/lymphocyte ratio; PLR, platelet/lymphocyte ratio; MPV, mean platelet volume.

### Elevated maternal white blood cells count in fetuses with enlarged ventricle group

**FIGURE 3:**
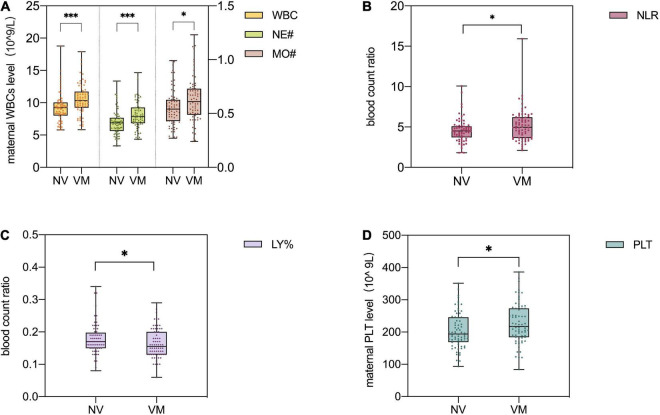
Different levels of inflammation markers in maternal peripheral blood in the normal ventricular group (*n* = 72) and fetal ventriculomegaly (VM) group (*n* = 70). Mann–Whitney *U* test was used to compare the non-parametric data **(A–C)** and Student *t*-test was used for parametric data **(D)**. **(A)** Comparison of maternal white blood cell (WBC), neutrophil (NE#), and monocytes (MO#) levels among different groups. The maternal WBC and NE# levels are shown by the left Y-axis of the image, and the MO# level is shown by the right Y-axis of the image. **(B)** Comparison of the maternal neutrophil/lymphocyte ratio (NLR) among different groups. **(C)** Comparison of the maternal lymphocyte percent (LY%) among different groups. **(D)** Comparison of maternal platelet count (PLT) levels among different groups. NV, normal ventricles; VM, ventriculomegaly; WBC, white blood cell; LY%, lymphocyte percent; NE#, neutrophil; MO#, monocytes; NLR, neutrophil/lymphocyte ratio; PLT, platelet count. ***P < 0.001, **P < 0.01, *P < 0.05. *P*-values less than 0.05 were considered statistically significant.

**FIGURE 4:**
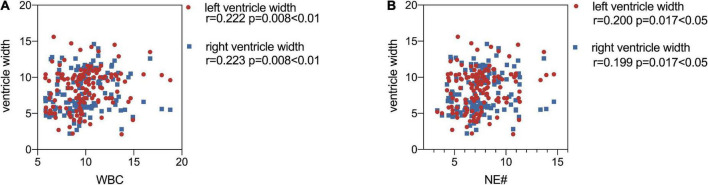
Scatter plots show the correlations of maternal white blood cell (WBC) **(A)**, and NE# **(B)** with left/right ventricle width. WBC, white blood cell; NE#, neutrophil. *P*-values less than 0.05 were considered statistically significant.

Maternal WBC, neutrophil (NE#), and monocytes (MO#) levels are shown in Table 2. As Figure 3A demonstrated, the level of maternal WBC in fetal VM was significantly higher than that in the normal ventricle group [10.320 (IQR = 9.248, 11.723) vs. 9.230 (IQR = 8.018, 10.050) 10^9/L; *P* < 0.001]. Similar to WBC, maternal NE# level in fetal VM was greatly higher than normal ventricle group [7.870 (IQR = 6.843, 9.260) vs. 6.895 (IQR = 5.640, 7.648) 10^9/L; *P* < 0.001]. Meanwhile, the level of MO# in fetal VM is also higher than control group [0.610 (IQR = 0.488, 0.730) vs. 0.540 (IQR = 0.430, 0.628) 10^9/L; *P* = 0.027]. Figure 4 showed the results of Spearman’s Rank Correlation. There were statistically significant and positive correlations between WBC and fetal left/right ventricular diameters (left: Spearman’s rho = 0.222; *P* = 0.008 < 0.01; right: Spearman’s rho = 0.223; *P* = 0.008 < 0.01). NE# was also significantly correlated with both left and right ventricular diameters (left: Spearman’s rho = 0.200; *P* = 0.017 < 0.05; right: Spearman’s rho = 0.199; *P* = 0.017 < 0.05). No correlation between ventricle width and other maternal analytes could be observed (Supplementary Table 1).

### Comparison of the levels of other clinical inflammation markers in the peripheral blood of the pregnancies

Neutrophil/lymphocyte ratio [4.965 (IQR = 3.618, 6.215) vs. 4.505 (IQR = 3.705, 5.088); *P* < 0.05] levels were higher in maternal with fetal VM compared to the normal ventricle group (Figure 3B). However, there are no significant group differences in the prevalence of maternal MLR and PLR levels. Besides, maternal lymphocyte percent (LY%) level in fetal VM group is clearly lower than normal ventricle group [0.155 (IQR = 0.130, 0.200) vs. 0.170 (IQR = 0.150, 0.198); *P* < 0.05; Figure 3C]. Similarly, the maternal PLTs were found to be 227.13 ± 62.278 in the fetal VM group, which was significantly higher than that of the normal ventricle group 204.810 ± 55.070 (*P* < 0.05; Figure 3D).

### Maternal white blood cells as early diagnostic markers in fetal ventriculomegaly

**FIGURE 5:**
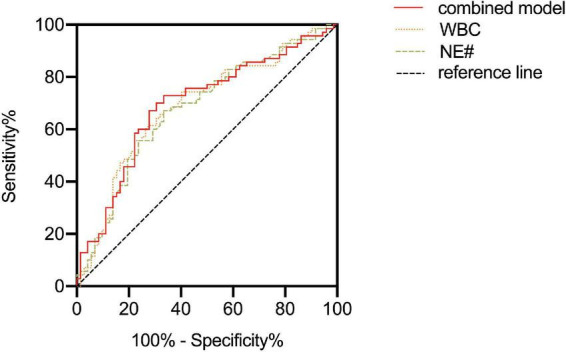
Receiver operating curves (ROC) characteristic of maternal white blood cell (WBC), NE#, and combined model were utilized to analyze the accuracy of maternal blood parameter levels to predict fetal ventriculomegaly (VM).

After adjusting the gestational age, WBC, NE#, MO#, NLR, and PLT were all promising predictors of fetal VM in the univariable logistic regression model (Table 3). As a continuous variable, WBC was associated with an enlarged ventricle diameter with an adjusted OR of 1.287 (95% CI = 1.097–1.511; *P* = 0.002). Based on the OR of LY% is 0.001, which is not statistically significant, LY% cannot be an effective index to predict fetal VM (*P* > 0.05). Fetal VM group was also associated with NE# (95% CI = 1.121–1.625; *P* = 0.002; adjusted OR = 1.350). However, in the multivariable model, only NE# was independently associated with fetal VM (95% CI = 1.123–1.624; *P* = 0.001; OR = 1.350), and the value of Hosmer–Lemeshow is 0.464 > 0.05 (Table 4). The diagnostic efficiency of these blood indexes was evaluated by the sensitivity, specificity, positive predictive values (PPV), negative predictive values (NPV), Youden index, and the area under the ROC curve (AUC; Table 5). Our results have shown that the diagnostic model accurately distinguishes fetuses with enlarged ventricles. Based on the ROC curve (Figure 5), the optimal cut-off value of maternal WBC levels as an indicator for the prediction of fetal VM was projected to be 9.400 × 10^9/L, which yielded a sensitivity of 74.3% and specificity of 59.7%, with the AUC at 0.688 (95% CI = 0.600–0.776; *P* < 0.001), and the NPV is 70.5%. As shown in Table 5, 7.205 × 10^9/L as a cut-off value for NE# (95% CI = 0.590–0.767) differentiated maternal with fetal VM from the normal fetal ventricle group with a sensitivity of 67.1%, a specificity of 66.7% (AUC = 0.749, *P* < 0.001). The cut-off value of maternal MO# levels was 0.665 × 10^9/L, and the positive predictive value was 71.4%, with an AUC of 0.608 (95% CI = 0.586–0.762; *P* < 0.05). PLT (AUC = 0.614; 95% CI = 0.521–0.707; *P* < 0.05) showed a greater discriminatory ability compared with NLR (AUC = 0.603; 95% CI = 0.509–0.698; *P* < 0.05). The combined analytes (WBC, NE#, MO#, NLR, and PLT) slightly improved the diagnostic effectiveness, with an AUC of 0.694 (*P* < 0.001). WBC has high sensitivity and high NPV to minimize missed diagnoses; MO# has high specificity and high PPV to minimize the number of false positive patients. Therefore, the biomarkers related to maternal inflammation are significantly increased in the fetal VM group, and maternal WBCs levels may be expected to be potential trait-related indicators of fetal VM.

**TABLE 3:** Predictors of fetal ventriculomegaly (VM) in univariable logistic regression model.

Variables	*B*	S.E.	Wald	*P*	OR	95% CI
WBC	0.253	0.082	9.554	0.002	1.287	1.097–1.511
LY%	−7.063	3.797	3.461	0.063	0.001	0.000–1.460
NE#	0.300	0.095	10.063	0.002	1.350	1.121–1.625
MO#	2.072	0.950	4.761	0.029	7.944	1.235–51.113
NLR	0.224	0.110	4.105	0.043	1.251	1.007–1.553
PLT	0.007	0.003	5.139	0.023	1.007	1.001–1.013

*P*-values less than 0.05 were considered statistically significant. WBC, white blood cell; LY%, lymphocyte percent; NE#, neutrophil; MO#, monocytes; NLR, neutrophil/lymphocyte ratio; PLT, platelet count; OR, odds ratio; 95% CI, confidence interval.

**TABLE 4:** Stepwise logistic regression results.

Variables	*B*	S.E.	Wald	*P*	OR	95% CI
NE#	0.300	0.094	10.158	0.001	1.350	1.123–1.624
Constant	–2.278	0.722	9.967	0.002	0.102	−

*P*-values less than 0.05 were considered statistically significant. OR, odds ratio; 95% CI, confidence interval; NE#, neutrophil.

**TABLE 5:** Performance of tested analytes as the best single under receiver–operator curve (ROC) analysis.

Parameters	Optimum cut-off	Se%	Sp%	PPV%	NPV%	AUC	95% CI
WBC	9.4	74.3	59.7	64.2	70.5	0.688	0.6–0.776
NE#	7.205	67.1	66.7	66.2	67.6	0.678	0.59–0.767
MO#	0.665	42.9	83.3	71.4	60.0	0.608	0.514–0.701
NLR	5.18	47.1	79.2	68.8	60.6	0.603	0.509–0.698
PLT	199.5	67.1	55.6	59.5	63.5	0.614	0.521–0.707
Combined model	0.465	72.9	66.7	68.0	71.7	0.694	0.607–0.782

AUC, area under the curve; Se, sensitivity; Sp, specificity; PPV, positive predictive values; NPV, negative predictive values; WBC, white blood cell; NE#, neutrophil; MO#, monocytes; NLR, neutrophil/lymphocyte ratio; PLT, platelet count.

## Discussion

This is the first study to evaluate the abnormal development of fetal brain structure by maternal blood parameters and fetal MRI during pregnancy. In this retrospective cohort study, we found that pregnancies with fetal VM have higher levels of WBC, MO#, NE#, NLR, and PLT compared with maternal with normal ventricle development fetuses. No significant associations were observed between fetal VM and maternal RBC, RGB, or HCT. In addition, logistic regression suggested that NE# counts might be useful to predict fetuses with enlarged lateral ventricles. Overall, these data suggest that elevated maternal blood cell levels as immunological biomarkers are linked to the abnormal fetal brain structure, especially fetal ventricle enlargement.

Previous studies have been conducted into the link between blood inflammatory biomarkers and neurodevelopmental disorders, particularly WBCs were significantly increased in schizophrenia and autism (Hesapcioglu et al., 2019; Jackson and Miller, 2020). For example, the research mentioned reduced gray matter and enlarged ventricles associated with the NE# count in first-episode psychosis and represents evidence of brain structure alteration associated with a dysregulated immune system (Núñez et al., 2019). Similarly, fetal enlarged ventricles are also related to maternal immune activation. The results of this explorative study suggest that maternal inflammatory blood cells including WBC, NE#, and MO# increased significantly in pregnancies with fetal VM. WBC levels have become a marker of systemic inflammatory response and are widely used in predicting neurodevelopmental disorders because of their inexpensive, widely used properties, and easy access to blood samples. Likewise, the current study detected a much higher NLR level in the fetal VM group. It is also noteworthy that elevated NLR levels are related to the inflammatory pathophysiology of neurodevelopmental disorders, and may become inflammation markers for attention-deficit hyperactivity disorder (Avcil, 2018). So future research should provide more evidence to support associating fetal VM with maternal NLR levels. In our study, PLT in the VM group was also significantly higher than that in the normal ventricle group. PLT is also regarded as an inflammatory marker in schizophrenia, and plays a pivotal role in its pathogenesis through the serotonin pathway (Ozdin and Boke, 2019). However, more forceful evidence is needed to reveal the potential relationship between maternal PLT level and fetal VM. Interestingly, no relationship was found between fetuses with enlarged lateral ventricles and maternal RBCs in the present study. Previous studies also considered that RBC level had little relationship with neurodevelopmental disorders, Semiz et al. (2014) have shown that RBC and HGB levels in patients with schizophrenia are similar to controls. In light of all these comparisons of blood parameters, we examined the efficacy of maternal inflammatory markers in distinguishing fetuses with VM by ROC analysis. Our study emphasizes that maternal blood parameters especially WBC and NE# counts had diagnostic effectiveness with AUC values ranging between 0.678 and 0.688. When combined these analytes (WBC, NE#, MO#, NLR, and PLT), the diagnostic effectiveness slightly improved with an AUC value of 0.694 (*P* < 0.001). In clinical practice, AUC values > 0.7 imply sufficient diagnostic effectiveness were able to distinguish patients accurately. However, the elevated maternal inflammatory markers still emphasized the importance of maternal immune dysregulation for fetal lateral ventricle development. More indicators and factors related to the mechanisms underlying fetal brain development need to be explored in the future.

Blood samples are obtained conveniently and help us understand the immune environment of fetal development. Pregnancies suffering from bacterial, viral, or parasite infections are characterized by abnormal levels of immune blood cells, as extensively discussed in a growing body of research, maternal immunologic biomarkers imbalance can induce negatively affecting on shaping neurodevelopmental trajectories, including regulating neuronal migration and neurogenesis, thus influencing short or long-lasting consequences in fetal postnatal neurodevelopment (Gumusoglu and Stevens, 2019; Cattane et al., 2020). Notably, previous studies have been conducted into the link between infection and fetal VM, for example, cytomegalovirus (CMV) infection is an important cause of fetal VM which affects nervous tissue development and leads to neurodevelopmental delay (Guo et al., 2021).

Other articles emphasize the importance of early brain structure altered as the determinant of neurodevelopmental disorder. Structural abnormalities are likely substrates for later neurocognitive impairment, and there is indeed compelling evidence that abnormal ventricles negatively impact fetal neurodevelopment delay (Fox et al., 2018; Thorup et al., 2019). The current study uses MRI to observe the fetal ventricle, which is the most common structural abnormality in the fetal stage and provides important information for fetal diagnosis and prognostication. Meanwhile, we considered that maternal blood index may serve as a potential biomarker for the early diagnosis of fetal VM. Our study emphasizes the vulnerability during the gestation stage when maternal immune dysregulation will interfere with fetal brain development.

Although our results reveal the association between maternal blood cell levels and abnormal fetal brain structure development, several limitations of the current study should be noted. First, this study is limited by the lack of data on neurodevelopmental outcomes. An issue for studies of fetal neurodevelopment is that outcome measures are often collected well after birth. We only demonstrated the relationship between fetal VM and maternal inflammatory markers without assessing the neurodevelopmental outcome of children. The clinical significance of these abnormal brain structure development remains to be evaluated by investigating the neurodevelopmental and cognitive outcomes of children in future follow-up studies. In addition, because of the retrospective nature of the study, body mass index, iron deficiency, history of smoking and alcohol use, and maternal stress-related hormones, which are considered as important confounding factors that influenced maternal blood parameters and fetal brain development, are not provided in the hospital database. Thus, these related variables are not tightly controlled. Besides, while the high WBCs counts are the signs of immunological abnormalities, the increase of the pro-inflammatory cytokines in peripheral blood levels also shows prenatal infections. In addition, as blood markers are dynamic and constantly changing indicators, repeated measurements may show small fluctuations, therefore long-term measurement of maternal blood markers during pregnancy may be considered in future studies, and more attention may be paid to individuals with abnormal indicators.

Although the presence of fetal brain structure abnormalities has been proven to be closely related to the neurodevelopmental delay and findings in fetal MRI can be used as an indication of the consequence, future research needs longitudinal analysis and exploration of mechanisms. A growing number of studies propose not only prenatal infections but also nutrition, stress, and antenatal maternal mood such as prenatal stress will also influence fetal neurodevelopment (Cattane et al., 2020; Nazzari and Frigerio, 2020). These conclusions suggest that multiple evaluations for fetal development are vital. To better diagnose and cope with abnormal fetal brain development in the early period, future studies need to consider inflammatory immune regulation, neurotransmitter secretion, nutritional metabolism, gene transcription, and other important factors which regulate fetal brain growth and neurodevelopment. Furthermore, valuation in the early years is also necessary for the detection of brain abnormalities, which could bring them timely rehabilitation and improve the neurological prognosis effectively. For individuals with neurodevelopmental defects, scientific intervention programs should be provided to minimize the adverse results caused by neurodevelopmental impairment. we believe that the results of this study will assist in evaluating the abnormal brain development in embryonic and inform current clinical management as well as parental counseling, particularly having important implications for the effective targeted intervention.

## Conclusion

In summary, the current study contributed to the emphasis on the importance of exposure to adverse environmental insults during critical periods of neurodevelopment as determinants of brain structure abnormalities. We demonstrated the significant differences in the levels of maternal inflammatory markers between fetuses with VM and fetuses with normal ventricles. High levels of maternal WBC, NE# counts, MO# counts, NLR, and PLTs were significantly increased in the fetal VM group. Logistic regression analyses suggested that NE# counts may be useful as novel markers to predict fetal VM. Studies reflect the importance of maternal infection for fetal brain growth, which could provide important information for prenatal diagnosis of CNS anomalies.

## Data availability statement

The original contributions presented in this study are included in the article/Supplementary material, further inquiries can be directed to the corresponding authors.

## Ethics statement

The studies involving human participants were reviewed and approved by The First Hospital of Jilin University. Written informed consent for participation was not required for this study in accordance with the national legislation and the institutional requirements. Written informed consent was obtained from the individual(s) for the publication of any potentially identifiable images or data included in this manuscript.

## Author contributions

QL, CL, and X-DJ were involved in study design and conceptualized. QL and X-WJ were responsible for collecting all samples and helping with statistical analysis. JX was involved in interpreting the data and intellectual input. JJ was involved in revising the draft. QL was involved in writing—original draft. CL and X-DJ were involved in editing the manuscript. All authors read and approved the final manuscript.
